# Association of PCSK9 plasma levels with metabolic patterns and coronary atherosclerosis in patients with stable angina

**DOI:** 10.1186/s12933-019-0949-3

**Published:** 2019-10-31

**Authors:** Chiara Caselli, Serena Del Turco, Rosetta Ragusa, Valentina Lorenzoni, Michiel De Graaf, Giuseppina Basta, Arthur Scholte, Raffaele De Caterina, Danilo Neglia

**Affiliations:** 1 CNR, Institute of Clinical Physiology, Via Moruzzi 1, 56100 Pisa, Italy; 2 Scuola Superiore Sant’Anna, Pisa, Italy; 3 Leiden University Medical Center, Leiden, The Netherlands; 4 Institute of Cardiology, University of Pisa, Pisa, Italy; 5 Fondazione Toscana G. Monasterio, Pisa, Italy

**Keywords:** PCSK9, Stable angina, Coronary atherosclerosis, Metabolic syndrome

## Abstract

**Objective:**

Aim of this study was to evaluate the relationship of plasma PCSK9 with metabolic and inflammatory profile and coronary atherosclerotic burden in patients with suspected CAD enrolled in the EVINCI study.

**Methods:**

PCSK9 was measured in 539 patients (60.3 ± 8.6 years, 256 males) with symptoms of CAD characterized by risk factors, bio-humoral profiles, and treatment. N = 412 patients underwent coronary computed tomography angiography (CTA) to assess the presence and characteristics of coronary atherosclerosis. A CTA score, combining extent, severity, composition, and location of plaques was computed.

**Results:**

Patients were divided according to PCSK9 quartiles: I (< 136 ng/mL), II–III (136–266 ng/mL), and IV quartile (> 266 ng/mL). Compared with patients in quartile IV, patients in quartile I had a higher prevalence of the metabolic syndrome and higher values of body mass index. LDL- and HDL-cholesterol were significantly lower in patients in the quartile I than in those in quartile IV. Coronary CTA documented normal vessels in 30% and obstructive CAD in 35% of cases without differences among PCSK9 quartiles. Compared with patients with the highest levels, patients with the lowest PCSK9 levels had a higher CTA score mainly due to higher number of mixed non-obstructive coronary plaques. At multivariable analysis including clinical, medications, and lipid variables, PCSK9 was an independent predictor of the CTA score (coefficient − 0.129, SE 0.03, P < 0.0001), together with age, male gender, statins, interleukin-6, and leptin.

**Conclusion:**

In patients with stable CAD, low PCSK9 plasma levels are associated with a particular metabolic phenotype (low HDL cholesterol, the metabolic syndrome, obesity, insulin resistance and diabetes) and diffuse non-obstructive coronary atherosclerosis.

*Trial registration* ClinicalTrials.gov NCT00979199. Registered September 17, 2009

## Background

Cardiovascular disease accounts for the largest proportion of deaths in Western countries [1]. Reduction in low-density lipoprotein (LDL) cholesterol, mainly with statins, has decreased the risk of cardiovascular events over the last few decades [2]. However, many individuals treated with statins do not achieve their target levels of LDL cholesterol, so that a residual risk associated with LDL levels may remain [3]. Proprotein convertase subtilisin/kexin type 9 (PCSK9), a protein involved in cholesterol homeostasis by enhancing degradation of the hepatic low-density lipoprotein receptor [4], has been recently identified as a new target for LDL lowering treatments. Gain of function mutations of PCSK9 are associated with high plasma LDL cholesterol levels and increased risk of cardiovascular events. On the other hand, loss of function mutations and treatment with PCSK9 inhibitors are consistently associated with lower risk of cardiovascular events, mainly attributed to relevantly decreased circulating LDL cholesterol [5].

The relationships between circulating PCSK9 levels, coronary disease and risk, is complex and might be partially independent of LDL cholesterol. PCSK9 plasma levels were associated with the severity of coronary lesions in patients with acute coronary syndrome and myocardial infarction [6–10]. Similarly, most studies exploring the association between plasma PCSK9 and early coronary atherosclerosis demonstrated a not clear direct relationship [11–18]. However, both the clinical presentation of coronary artery disease (CAD) is changing over time and some risk factors such as obesity, diabetes and metabolic syndrome are emerging as major disease determinants in current populations [19, 20]. There are only few clinical studies evaluating the relationships between circulating levels of PCSK9, other circulating determinants of the atherosclerotic risk and a comprehensive description of coronary artery disease (CAD) phenotype [18]. Studies in this area may be useful to gain new insights on regulatory mechanisms of PCSK9 expression and its effects on CAD in contemporary populations.

The main purpose of the present study was to evaluate the interplay of circulating PCSK9 levels with markers of coronary atherosclerosis processes and disease in a contemporary population of patients with stable angina enrolled in the EValuation of INtegrated Cardiac Imaging (EVINCI) study [21]. The cardiovascular phenotype of the EVINCI patients has been well described by extensive clinical and bio-humoral profiling and cardiac imaging [22–26]. In particular, the global coronary atherosclerotic burden could be evaluated at the patient level using a comprehensive coronary computed tomography angiography (CTA) score [27, 28]. Taking advantage of these peculiarities, specific objectives of the present study were to assess the association of PCSK9 plasma levels with clinical risk factors and bio-humoral profiles, including metabolic and inflammatory biomarkers, as determinants of presence, extent and severity of coronary atherosclerosis, beyond LDL cholesterol levels.

## Materials and methods

### Study design and population

Design and primary results of the EVINCI study have been previously published [21]. In brief, EVINCI was a multicenter, multinational, prospective, observational study that included 697 patients with stable chest pain or equivalent symptoms and intermediate probability of CAD from 14 centres in 7 European countries (http://www.clinicaltrials.gov, NCT00979199). The recruitment period was 39 months (March 2009 to June 2012). According to the protocol, each patient underwent coronary CTA, stress imaging by myocardial perfusion imaging or wall motion imaging. If at least one of non-invasive tests was positive, invasive coronary angiography was performed. Blood samples were collected from patients in fasting state during the first enrolment visit, before non-invasive imaging, and plasma aliquots were stored in the EVINCI Bio-Bank. Ethics Committee approval was provided by each participating center, and all subjects provided written informed consent.

**Fig. 1:**
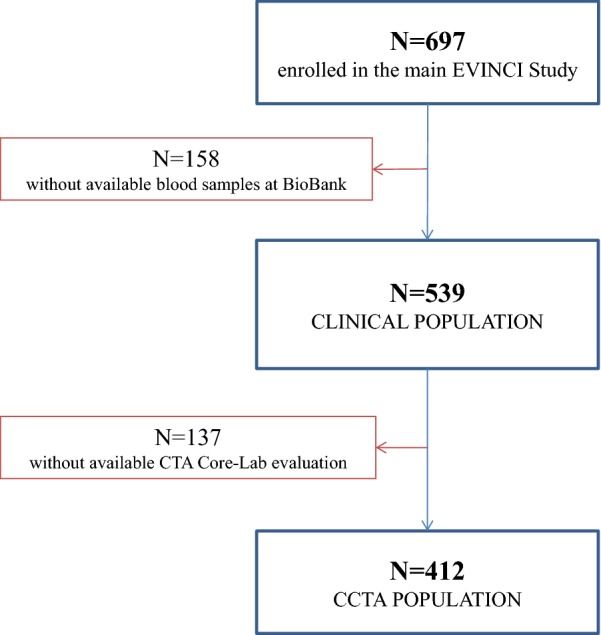
Study flow diagram. After exclusions, 539 subjects had a completed clinical profile and available blood samples for PCSK9 testing. In this clinical sample, 412 patients had available CTA

For the purposes of the present analysis, plasma levels of PCSK9 were evaluated in patients from the EVINCI study who had a completed clinical profile and available blood samples stored in the BioBank (clinical population). Further analyses were performed in the subgroup of patients with available coronary CTA core laboratory analyses (CTA population). The study flow diagram is detailed in Fig. 1.

### Anthropometric measures and clinical definitions

Height and body weight were measured by standard methods with the participants in light clothing. Body mass index (BMI) was calculated as body weight (in kg) divided by the square of the height (in m). Homeostatic model assessment of insulin resistance index (HOMA-IR) was calculated as fasting glucose (mg/dL) × fasting insulin (pmol/L)/8.66 [29]. Presence and extent of obesity was defined according to body mass index (BMI) categories [30]. Diabetes was defined as fasting plasma glucose (FPG) > 125 mg/dL or treatment. The presence of metabolic syndrome was diagnosed according to the National Cholesterol Education Program Adult Treatment Panel III [31] criteria, when three or more of the following criteria were present:

1. Raised triglyceride level: ≥ 1.7 mmol/L (150 mg/dL);
2. Reduced HDL-cholesterol: < 1.03 mmol/L (40 mg/dL) in male and < 1.29 mmol/L (50 mg/dL) in female;
3. Raised blood pressure (systolic blood pressure ≥ 130 or diastolic blood pressure ≥ 85 mmHg) (or treatment of previously diagnosed hypertension);
4. Raised fasting plasma glucose [FPG ≥ 5.6 mmol/L (100 mg/dL)] (or previously diagnosed type 2 diabetes);
5. Central obesity (BMI ≥ 30 kg/m^2^).

Since data on waist circumference were not collected during the EVINCI study, central obesity was defined as BMI over 30 kg/m^2^, based on the assumption that in this condition waist circumference does not need to be measured [32].

### PCSK9 measurements and bio-humoral profile

PCSK9 plasma levels were measured in blood samples stored in the EVINCI biological bank at the Consiglio Nazionale delle Ricerche (CNR)-Institute of Clinical Physiology, Pisa, Italy) [20, 22] by a dedicated ELISA (Quantikine ELISA, R&D Systems) (0.07 ± 0.007 ng/L LoD; 3.96% intra-run variation; 8.33% inter-run variation). Additional specific metabolic and inflammatory biomarkers were measured using standard methods [24]. LDL cholesterol was calculated according to Friedewald formula [33].

### Coronary CTA

The methodology for coronary CTA acquisition and analysis in the EVINCI study has been previously reported in detail [21, 23, 24]. Briefly, each segment of the AHA 17-coronary segment model was assessed for interpretability, and interpretable segments were evaluated for the degree of stenosis of the coronary artery. If a plaque was present, plaque composition was visually determined (calcified, non calcified, and mixed). Only one type of plaque composition could be assigned to a single segment. A previously validated CTA score, used as an indicator of the global coronary atherosclerotic burden and risk, was derived in each patient by integration of all data on the location, severity and composition of CAD [27, 28].

### Statistical analysis

Categorical variables are presented as numbers (percentage), continuous variables as mean ± SD. The logarithmic transformation of continuous variables was used in the analyses. To better discriminate patients with the highest and the lowest PCSK9 plasma levels, clinical, biohumoral and imaging features were compared among 4 groups according to PCSK9 quartiles (Additional file 1: Tables S1, S2, S3) or among 3 groups (I vs II–III vs IV quartiles) using analysis of variance (ANOVA) with post hoc tests for multiple comparisons or the Chi square test, as appropriate. Comparison between two groups was performed using the Student’s *t* test. Pearson correlation was used to assess the relation between bio-humural variables in specific patients subgroups.

Multivariate linear regression was used to estimate the effect of clinical and bio-humoral variables, including PCSK9 plasma levels, on the CTA score. A multivariable model was developed, considering variables with a P value < 0.1 at univariable analysis, and then using backward and forward stepwise selections to build-up the final model. All analyses were performed using the SPSS 23 software. A 2-sided value of P < 0.05 was considered statistically significant. There is no multiplicity adjustment implemented in statistical testing.

## Results

### Relationships between PCSK9 concentrations, clinical and bio-humoral characteristics

The clinical population consisted of 539 EVINCI patients with a completed clinical and bio-humoral profile, and in whom PCSK9 plasma levels were determined (Fig. 1). The mean value of PCSK9 was 212.0 ng/mL (SD 104.9 ng/mL), and the median value was 183.8 ng/mL (95% CI 203.2–220.9 ng/mL).

Clinical characteristics among different PCSK9 groups are detailed in Table 1. Patients in the highest PCSK9 quartile had a more frequent family history of CAD and a lower BMI. On the other hand, metabolic syndrome was more prevalent in the lowest Quartile and was progressively less frequent from Quartile I to IV. Among medications, the use of anti-diabetic drugs and aspirin, but not of statins, varied significantly among groups.

**Table 1:** Clinical characteristics of the clinical population relative to PCSK9 groups

	Clinical population n = 539	Quartile I < 138 ng/L n = 135	Quartile II–III 138–264 ng/L n = 270	Quartile IV > 264 ng/L n = 134	P value
Demographics					
Age, years	60 ± 9	61 ± 9	60 ± 9	61 ± 8	ns
Male gender	326 (60)	88 (65)	166 (61)	72 (53)	0.1411
Clinical characteristics					
Typical angina	140 (26)	30 (22)	66 (24)	44 (33)	ns
Atypical angina	321 (60)	78 (58)	166 (61)	77 (57)	
Non-anginal chest pain	78 (14)	27 (20)	38 (14)	13 (10)	
LVEF%	60 ± 8	60 ± 9	60 ± 9	61 ± 7	ns
Pre-test probability of CAD	48 ± 19	48 ± 18	48 ± 19	49 ± 20	ns
Cardiovascular risk factors					
Family history of CAD	189 (35)	40 (30)	90 (33)	59 (44)	0.0328
Diabetes	160 (30)	37 (27)	90 (33)	33 (25)	ns
Hypercholesterolemia	322 (60)	77 (57)	163 (60)	82 (61)	ns
Hypertension	360 (67)	88 (65)	181 (67)	91 (68)	ns
Smoking	133 (25)	30 (22)	69 (26)	34 (25)	ns
BMI, kg/m^2^	27.7 ± 4.3	27.9 ± 4.0	28.0 ± 4.3	26.8 ± 4.6	0.0282
Metabolic syndrome	185 (34)	54 (40)	100 (37)	31 (23)	0.0059
Pharmacological therapies					
Beta-blockers	215 (40)	64 (47)	105 (39)	46 (34)	ns
Calcium channel blockers	74 (14)	21 (16)	32 (12)	21 (16)	ns
ACE inhibitors	166 (31)	43 (32)	87 (32)	36 (27)	ns
ARBs	91 (17)	23 (17)	43 (16)	25 (19)	ns
Diuretics	93 (17)	27 (20)	44 (16)	22 (16)	ns
Anti-diabetic	111 (21)	27 (20)	66 (24)	18 (13)	0.0354
Statins	279 (52)	72 (53)	148 (55)	59 (44)	ns
Aspirin	316 (59)	94 (70)	147 (54)	75 (56)	0.0107
Anti-coagulants	11 (2)	2 (1)	5 (2)	4 (3)	ns

Continuous variables are presented as mean ± standard deviation, categorical variables as absolute N and (%)

**Fig. 2:**
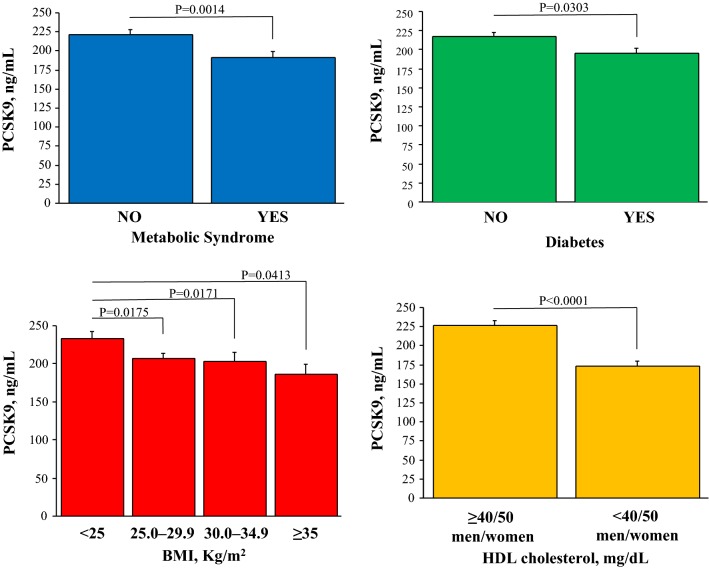
PCSK9 plasma levels in patients subdivided according to the presence of metabolic syndrome or diabetes, and according to BMI and HDL cholesterol classes

**Fig. 3:**
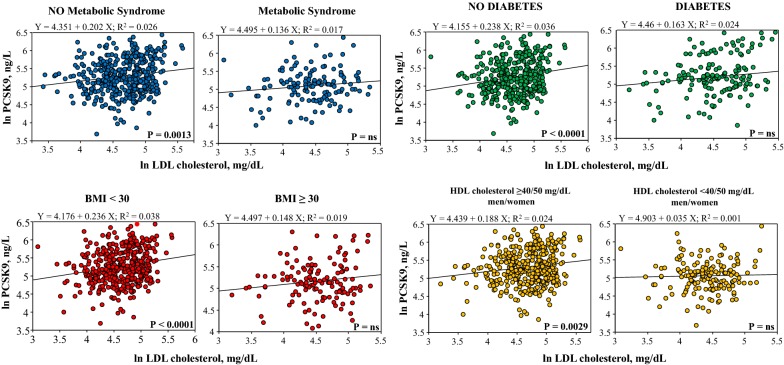
Relationship between circulating levels of PCSK9 and LDL cholesterol in patients with/without the metabolic syndrome, diabetes, obesity, and low HDL cholesterol

The bio-humoral profile comparison among the various PCSK9 groups are reported in Table 2. There was a significant increasing trend in circulating transaminases, alkaline phosphatases, total cholesterol, LDL cholesterol, Apo B and Lipoprotein (a) passing from the lowest to the highest PCSK9 quartiles. Conversely, HDL cholesterol, total/HDL cholesterol ratio, Apo A1 and adiponectin levels were lower, and insulin levels, HOMA-IR and HO-1 higher, in the lowest PCSK9 quartile. No differences were observed for inflammatory biomarkers among groups. In the overall population, PCSK9 levels were significantly lower in patients with the metabolic syndrome, or diabetes, or high BMI, or low HDL cholesterol (Fig. 2). The correlation of PCSK9 with LDL cholesterol plasma levels was lost in these specific patients groups (Fig. 3).

**Table 2:** Bio-humoral characteristics of the clinical population

	Clinical population n = 539	Quartile I < 138 ng/L n = 135	Quartile II–III 138–264 ng/L n = 270	Quartile IV > 264 ng/L n = 134	P value
Renal					
Creatinine, mg/dL	0.87 ± 0.23	0.91 ± 0.27	0.87 ± 0.22	0.86 ± 0.19	ns
Hepatic					
AST, IU/L	24 ± 10	24 ± 11	24 ± 10	26 ± 9	0.0411
ALT, IU/L	21 ± 13	19 ± 11	21 ± 14	23 ± 13	0.0180
ALP, IU/L	51 ± 18	47 ± 17	49 ± 16	61 ± 20	< 0.0001
Metabolic (glucose)					
FTP, mg/dL	112 ± 36	109 ± 30	116 ± 41	109 ± 29	ns
FTP ≥ 100 mg/dL^a^	339 (63)	82 (61)	174 (64)	83 (62)	ns
Insulin, μUI/mL	11.6 ± 11.0	13.3 ± 12.5	11.3 ± 10.1	10.3 ± 10.1	0.0345*
HOMA-IR index	3.5 ± 4.2	3.9 ± 4.5	3.5 ± 4.1	2.9 ± 3.2	0.0569*
Metabolic (lipid)					
Total cholesterol, mg/dL	183 ± 49	171 ± 43	180 ± 45	201 ± 55	< 0.0001
LDL, mg/dL	106 ± 40	100 ± 37	104 ± 38	118 ± 45	0.0015
HDL, mg/dL	52 ± 17	46 ± 13	52 ± 15	61 ± 19	< 0.0001
HDL ≤ 40 mg/dL, male or HDL ≤ 50 mg/dL, female^a^	160 (30)	60 (44)	81 (30)	19 (14)	< 0.0001
Total/HDL cholesterol	3.7 ± 1.1	3.8 ± 1.2	3.7 ± 1.2	3.5 ± 1.1	0.0193
Apo A1, mg/dL	143 ± 32	132 ± 32	143 ± 30	154 ± 34	< 0.0001
Apo B, mg/dL	87 ± 28	82 ± 28	87 ± 27	93 ± 29	0.0024
Lipoprotein (a)	21.3 ± 23.2	15.8 ± 18.2	21.7 ± 23.4	24.9 ± 26.3	0.0018
Triglicerides, mg/dL	124 ± 81	126 ± 84	127 ± 86	118 ± 68	ns
Triglyceride ≥ 150 mg/dL^a^	131 (24)	34 (25)	67 (25)	30 (23)	ns
Inflammatory and vascular					
hs-CRP, mg/dL	0.40 ± 1.09	0.41 ± 0.61	0.38 ± 1.35	0.43 ± 0.81	ns
Interleukin 6, ng/L	1.35 ± 2.35	1.56 ± 2.71	1.27 ± 2.45	1.30 ± 1.67	ns
Adipokines					
Adiponectin, μg/mL	9.8 ± 6.9	8.11 ± 5.1	9.98 ± 7.2	11.19 ± 7.6	0.0006
Leptin, ng/mL	9.93 ± 10.66	11.38 ± 13	9.16 ± 9	9.99 ± 10.98	ns

Continuous variables are presented as mean ± standard deviation

* I vs. IV quartile

^a^Criteria of metabolic syndrome

### Relationships between PCSK9 concentrations, statin use and lipids

**Fig. 4:**
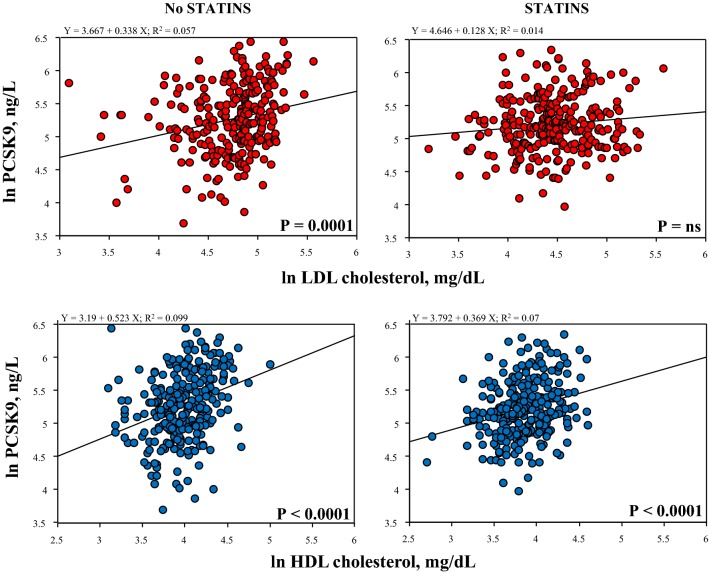
Effects of statin use on the relationships between PCSK9 and LDL and HDL cholesterol

Statin treatment did not significantly affect PCSK9 plasma levels (222 ± 1 ng/mL vs 202 ± 9 ng/mL, no statin users vs statin users, P = ns). Moreover, statin treatment abolished the significant correlation between PCSK9 and LDL cholesterol, while had no effect on the tight correlation of PCSK9 levels with HDL cholesterol (Fig. 4).

### Relationship between PCSK9 concentrations and CAD

**Fig. 5:**
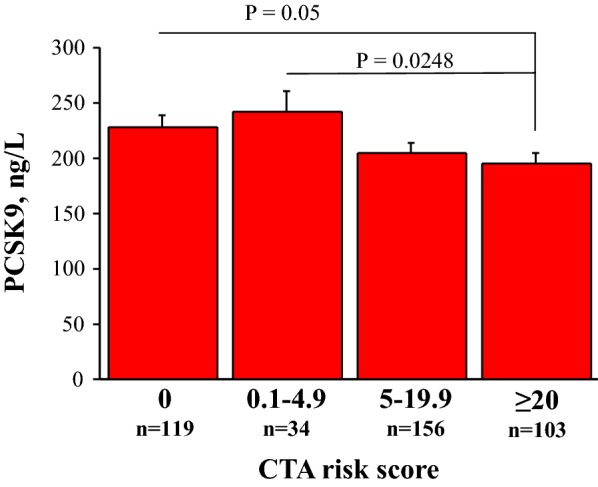
PCSK9 plasma levels according to CTA score categories

PCSK9 plasma levels were not associated with obstructive CAD at coronary CTA (Table 3). In fact, the diagnosis of obstructive CAD (i.e., at least one plaque with either > 50% or > 70% diameter stenosis) and the number of obstructive plaques per patient were similar across PCSK9 groups. On the other hand, PCSK9 plasma levels were inversely related with non obstructive CAD and the extent of global coronary atherosclerotic burden. In fact, the number of non-obstructive plaques and in particular of mixed plaques, as well as the global CTA score were significantly higher in patients with the lowest PCSK9 levels (Quartile I) as compared with those with the highest levels (Quartile IV). In the whole population, PCSK9 plasma levels showed a significant trend to reduction among CTA score classes, in particular in patients with CTA score > 5 (Fig. 5).

**Table 3:** Angiography imaging results relative to PCSK9 groups

	CTA population (n = 412)	Quartile I < 136 ng/L n = 103	Quartile II–III 136–266 ng/L n = 206	Quartile IV > 266 ng/L n = 103	P value
Obstructive CAD at CTA					
Stenosis > 50%	144 (35)	40 (39)	71 (34)	33 (32)	ns
Stenosis > 70%	45 (11)	12 (12)	23 (11)	10 (10)	ns
Coronary plaques					
Total N. of plaques	3.9 ± 3.8	4.36 ± 3.89	3.67 ± 3.77	3.37 ± 3.77	0.0621*
N. of non-obstructive plaques	3.0 ± 3.1	3.33 ± 2.89	3.14 ± 3.10	2.42 ± 2.93	0.0322*
N. of obstructive plaques	0.9 ± 1.7	0.94 ± 1.57	0.73 ± 1.42	0.85 ± 1.87	ns
N. of calcified plaques	0.9 ± 1.7	0.85 ± 1.84	0.93 ± 1.69	0.81 ± 1.74	ns
N. of mixed plaques	2.6 ± 3.2	3.14 ± 3.42	2.49 ± 3.12	2.19 ± 3.15	0.0356*
N. of non-calcified plaques	0.5 ± 0.9	0.38 ± 0.85	0.54 ± 1.02	0.38 ± 0.72	ns
CTA score	12 ± 11	13.17 ± 11.53	11.83 ± 10.71	9.99 ± 11.11	0.0391*

Continuous variables are presented as mean ± standard deviation, categorical variables as absolute N and (%)

* I vs. IV quartile

At univariable and multivariable analyses, including all clinical (Additional file 1: Table S4) and bio-humoral variables (Additional file 1: Table S5), low PCSK9 levels were an independent predictor of the CTA score together with age, gender, interleukin-6 and leptin (Table 4). Interestingly, when HDL cholesterol plasma levels were forced into the final multivariable model, the association of PCSK9 with the CTA score lost statistical significance (Table 4).

**Table 4:** PCSK9 and CTA score at multivariate analysis

	CTA score Unadjusted model			CTA score HDL cholesterol adjusted model		
	Coefficient	SE	P value	Coefficient	SE	P value
Age	0.070	0.006	< 0.0001	0.069	0.006	< 0.0001
Male gender	0.719	0.131	< 0.0001	0.631	0.139	< 0.0001
Statin	0.406	0.113	0.0004	0.380	0.114	0.0010
Interleukin 6	0.267	0.119	0.0186	0.266	0.119	0.0256
Leptin	− 0.190	0.069	0.0066	− 0.213	0.070	0.0026
PCSK9	− 0.267	0.113	0.0186	− 0.218	0.118	0.0660
HDL cholesterol	–	–	–	− 0.347	0.204	0.0670

## Discussion

In a European population of patients with low prevalence of obstructive CAD, the present study demonstrated that higher levels of circulating PCSK9, while being related with moderately higher levels of total and LDL cholesterol, were not associated with obstructive coronary disease. On the other hand, the novel finding of this study was that lower PCSK9 was associated with specific metabolic abnormalities and diffuse coronary atherosclerosis, beyond LDL cholesterol levels and statin use. In particular patients with lower PCSK9 had low HDL cholesterol, a higher CTA score (an integrated measurement of coronary atherosclerosis and risk) and a higher number of non-obstructive and mixed coronary atherosclerotic plaques. At multivariable analyses, lower PCSK9 levels were an independent determinant of the CTA score, and HDL cholesterol significantly contributed to this association.

### PCSK9 and coronary atherosclerotic disease

The relationship between circulating PCSK9 levels and CAD has been extensively explored. PCSK9 plasma levels have been associated with obstructive coronary lesions in patients with acute coronary syndrome and myocardial infarction [6–10] as well as with subclinical coronary atherosclerosis and imaging markers of atherosclerotic risk even if in some reports this relationship is not so clear [11–18]. The association of PCSK9 with coronary atherosclerosis in contemporary populations may be influenced by a number of factors. There is a shift in the clinical presentation of atherosclerosis from acute coronary syndromes to chronic atherosclerotic disease, which implies a possible change in the underlying mechanisms and prevents a simple extension of the results obtained by association studies in high risk patients to lower risk populations [19]. The diffusion of treatments with lipid lowering drugs have altered the relationship between lipid profiles and coronary disease [19, 20] and may uncover other emerging disease determinants, such as obesity, metabolic syndrome and diabetes. The present study was specifically designed to explore the relationships between circulating levels of PCSK9, comprehensive circulating determinants of the atherosclerotic risk and precise characterization of coronary artery disease in contemporary patient populations with suspected CAD. We did not find an association of PCSK9 levels with obstructive CAD but we documented a clear inverse correlation between PCSK9 and global coronary atherosclerotic burden as described by CTA score. These somewhat surprising results could be explained tacking into account some peculiarity of the EVINCI study. Firstly, the population was a contemporary European multicentric group of patients with stable angina, receiving optimal medical treatment and with a lower prevalence of obstructive CAD (35% of patients with CTA stenosis > 50% and 11% with stenosis > 70%) than usually found either in patients with acute coronary syndromes [7, 8] or referred to coronary catheterization in whom circulating PCSK9 had been previously measured [9, 10]. Secondly, using a comprehensive CTA score, we were able to assess the global coronary atherosclerotic burden at the patient level [27, 28]. In fact, this score, incorporating number and characteristics of all coronary plaques, is a better marker of the presence and extent of coronary atherosclerosis than estimates of CAD presence and severity only based on “hemodynamically significant” lesions. Thus, the CTA score takes into account the possible effects of multiple atherogenic processes on the final coronary phenotype and is a comprehensive predictor of coronary outcome [28]. This was particularly relevant considering that the present study group had a peculiar metabolic profile linked to diffuse coronary atherosclerosis in the absence of high LDL cholesterol levels usually associated with obstructive CAD.

### PCSK9 and metabolic profile

In our population circulating PCSK9 was still correlated with total and LDL cholesterol [together with Apo B and Lipoprotein (a)] but showed also a strong correlation with HDL cholesterol levels (together with Apo A1). The relationship of PCSK9, total or LDL cholesterol and Lipoprotein (a) has been demonstrated in many different populations and in large study cohorts [11, 34–37]. On the other hand, the relationship of PCSK9 levels and HDL cholesterol is less well established, and a clear involvement of PCSK9 in HDL metabolism is indeed uncertain [12, 38–42]. Interestingly, we found that the relationships of PCSK9 with LDL cholesterol was affected and, as matter of fact, obscured by statin treatment, while that with HDL cholesterol was not (Fig. 4). This is consistent with previous observations studying the effects of statin treatment on PCSK9 levels [43–45] showing that the direct correlation between PCSK9 and LDL cholesterol is lost after treatment [43]. Current results add evidence that statins do not affect the strong relationship of PCSK9 with HDL cholesterol.

We found a relationship of low PCSK9 levels with insulin resistance or diabetes, obesity or metabolic syndrome. The association of PCSK9 with glucose metabolism has been extensively studied at epidemiological, genetic, clinical and animal levels [46–50]. The possible interaction of PCSK9, glucose metabolism and treatment has also been investigated. Some antidiabetic drugs have been shown to decrease PCSK9 levels [51] while treatment with PCSK9 inhibitors was not associated with increased incidence of diabetes [52]. Even if most findings suggest a direct correlation of PCSK9 with abnormal glucose metabolism and insulin resistance, some studies suggest an opposite relation similar to that found in the present work.

In particular, patients with specific loss-of-function PCSK9 mutations or polymorphisms had increased prevalence in pre-diabetes and diabetes [53] as well as higher HOMA-IR index and insulin levels [54]. Interestingly, the direct relationship between PCSK9 and Lipoprotein (a) observed in the present study is in keeping with the association of low PCSK9 levels with insulin resistance and diabetes. In fact, in a large population study and metanalysis Paige et al. demonstrated the association of low Lipoprotein (a) concentrations and higher risk of type-2 diabetes [55].

Similarly, the inverse correlation of circulating PCSK9 with BMI, consistent with the direct correlation with adiponectin, observed in the present study, was not a completely expected result [46, 56]. Actually, an inverse association between PCSK9 and BMI had been already observed in some low cardiovascular risk populations such as healthy subjects [11], patients with an altered metabolic liver function, such as those with an increased hepatic fat [57], children and adolescents after adjustment for age, glucose, insulin, and adiponectin [58]. However, the mechanism of these associations is still unknown.

Similarly, clinical, genetic, and experimental evidence linked PCSK9 with metabolic syndrome [46]. In the present study, we found no association between PCSK9 and some components of the metabolic syndrome such as triglycerides and hypertension, while we found a very strong association between low PCSK9 levels and HDL cholesterol levels, independent of statin use. The association between lower PCSK9 levels and lower levels of adiponectin further underlines the link of PCSK9 with a specific metabolic phenotype [56]. As matter of fact, these results add some complexity to the known relation between PCSK9 and cholesterol. In fact, at least in this population, diabetes, obesity and the metabolic syndrome seem to override the influence of PCSK9 on LDL metabolism, as demonstrated by the loss of the correlation between PCSK9 and LDL cholesterol in patients with altered metabolic phenotype (Fig. 3).

Moreover, we also observed a tight relationship between circulating PCSK9 and liver enzymes, classically considered as markers of liver cellular damage. More recently liver enzymes have been also considered as surrogate biomarkers of liver metabolic function [59]. In the present population liver enzymes where in the normal range and the observed correlation with PCSK9 might express variable liver metabolic function (Additional file 1: Tables S6, S7). This observation needs further validation and the molecular mechanisms could be explored.

### PCSK9, metabolic phenotype, and coronary atherosclerosis

Given the peculiar metabolic patterns associated with low PCSK9 levels and the inverse relationship observed between PCSK9 and the CTA score, the present results suggest a possible pathophysiologic link between low circulating PCSK9, a metabolic phenotype characterized in particular by low HDL cholesterol and diffuse coronary atherosclerosis in a population at low prevalence of obstructive CAD. In a previous study performed in this population, we demonstrated that low plasma HDL cholesterol was an independent predictor of the CTA score together with age, gender, leptin and IL6 [23]. In the present study, low PCSK9 was an independent predictor of the CTA score in a multivariable model including the same variables and outperforming a wide variety of risk factors and bio-humoral markers. However, when HDL cholesterol levels were added to the analysis, the association of PCSK9 with the CTA score lost significance, strongly suggesting a contribution of HDL cholesterol to PCSK9-associated atherosclerotic risk. It has been very recently reported that individuals who reach low levels of LDL cholesterol may be persistently at high risk of atherosclerotic disease when total/HDL cholesterol ratio is discordantly higher [60]. Patients with such discordance had a greater atherogenic clinical profile with high levels of triglycerides, high BMI and a high prevalence of risk factors, including diabetes [60]. Interestingly, a similar metabolic pattern was recognized in the present study in patients with low PCSK9 levels who also showed low HDL and high total/HDL cholesterol ratio (Table 2).

## Limitation

This study has some limitation. PCSK9 is genetically regulated, but a genetic testing was not included in the present study. Moreover, experimental evidences demonstrate that PCSK9 is compartimentalized within the plasma, with almost 40% of PCSK9 bound to LDL and Lipoprotein (a) particles, and the remainder not associated with apolipoprotein B-containing lipoproteins [18], but the methods used in this study could not assess these interactions. The associations described here were obtained in a population with relatively low levels of PCSK9, total and LDL cholesterol and low prevalence of obstructive CAD and cannot be directly extended to wider populations with more variable bio-humoral, metabolic and CAD profiles. In our study, circulating PCSK9 was measured only at the time of enrolment, when 52% of patients were already under statin treatment. Specific data on type, duration and dose of current statin or other lipid lowering treatment were not collected in the EVINCI study. Thus, the effect of statins or other lipid lowering drugs on PCSK9 plasma levels in the same patients could not be directly evaluated due to the lack of a paired comparison before and after treatment. Moreover, it is not possible to directly extend these findings to pharmacological inhibition of PCSK9 via monoclonal antibody or short interfering RNA methods.

## Conclusion

This study extends knowledge of the relationships of PCSK9 with glucose and lipid metabolism and coronary atherosclerosis in patients with stable angina and low prevalence of obstructive CAD. Based on the present results, it can be speculated that in such low risk population there could be a link between lower levels of PCSK9, a particular metabolic phenotype (low HDL cholesterol, the metabolic syndrome, obesity, insulin resistance and diabetes) and the propensity to develop diffuse coronary atherosclerosis. Other studies are needed to confirm this association and explore its possible pathophysiologic mechanisms and prognostic implications. From a clinical perspective, the new information gathered on the interaction of PCSK9 individual profiles with glucose and lipid metabolic status and the atherosclerotic phenotype might be useful to target individual treatment in the context of a personalized medicine approach.

## Supplementary information

**Additional file 1.** Additional tables.

## Data Availability

The data that support the findings of this study are available from FTGM and IFC-CNR but restrictions apply to the availability of these data, which were under authorization for the current study and in compliance with GDPR 2016/679, and so are not publicly available. Data are however available from the corresponding author on reasonable request and with permission of FTGM and IFC-CNR.

## Abbreviations

BMI: body mass index
CAD: coronary artery disease
CTA: computed tomography angiography
EVINCI: EValuation of INtegrated Cardiac Imaging
FPG: fasting plasma glucose
HDL: high-density lipoprotein
HOMA-IR: homeostatic model assessment of insulin resistance index
LDL: low-density lipoprotein
PCSK9: proprotein convertase subtilisin/kexin type 9

## References

[1] Roth GA, Johnson C, Abajobir A (2017). Global, regional, and national burden of cardiovascular diseases for 10 causes, 1990 to 2015. J Am Coll Cardiol.

[2] Baigent C, Keech A, Kearney PM, Blackwell L, Buck G, Pollicino C, Kirby A, Sourjina T, Peto R, Collins R, Simes R, Cholesterol Treatment Trialists’ (CTT) Collaborators (2005). Efficacy and safety of cholesterol-lowering treatment: prospective meta-analysis of data from 90,056 participants in 14 randomised trials of statins. Lancet.

[3] Shimada YJ, Cannon CP (2015). PCSK9 (Proprotein convertase subtilisin/kexin type 9) inhibitors: past, present, and the future. Eur Heart J.

[4] Allard D, Amsellem S, Abifadel M, Trillard M, Devillers M, Luc G, Krempf M, Reznik Y, Girardet JP, Fredenrich A, Junien C, Varret M, Boileau C, Benlian P, Rabès JP (2005). Novel mutations of the PCSK9 gene cause variable phenotype of autosomal dominant hypercholesterolemia. Hum Mutat.

[5] Cohen JC, Boerwinkle E, Mosley TH, Hobbs HH (2006). Sequence variations in PCSK9, low LDL, and protection against coronary heart disease. N Engl J Med.

[6] Pott J, Schlegel V, Teren A, Horn K, Kirsten H, Bluecher C, Kratzsch J, Loeffler M, Thiery J, Burkhardt R, Scholz M (2018). Genetic regulation of PCSK9 (Proprotein Convertase Subtilisin/Kexin Type 9) plasma levels and its impact on atherosclerotic vascular disease phenotypes. Circ Genom Precis Med..

[7] Cariou B, Guérin P, Le May C, Letocart V, Arnaud L, Guyomarch B, Pichelin M, Probst V (2017). Circulating PCSK9 levels in acute coronary syndrome: results from the PC-SCA-9 prospective study. Diabetes Metab..

[8] Bae KH, Kim SW, Choi YK, Seo JB, Kim N, Kim CY, Lee WK, Lee S, Kim JG, Lee IK, Lee JH, Park KG (2018). Serum levels of PCSK9 are associated with coronary angiographic severity in patients with acute coronary syndrome. Diabetes Metab J..

[9] Cheng JM, Oemrawsingh RM, Garcia-Garcia HM, Boersma E, van Geuns RJ, Serruys PW, Kardys I, Akkerhuis KM (2016). PCSK9 in relation to coronary plaque inflammation: results of the ATHEROREMO-IVUS study. Atherosclerosis..

[10] Almontashiri NA, Vilmundarson RO, Ghasemzadeh N, Dandona S, Roberts R, Quyyumi AA, Chen HH, Stewart AF (2014). Plasma PCSK9 levels are elevated with acute myocardial infarction in two independent retrospective angiographic studies. PLoS ONE.

[11] Zhu YM, Anderson TJ, Sikdar K, Fung M, McQueen MJ, Lonn EM, Verma S (2015). Association of proprotein convertase subtilisin/kexin type 9 (PCSK9) with cardiovascular risk in primary prevention. Arterioscler Thromb Vasc Biol.

[12] Nose D, Shiga Y, Ueda Y, Idemoto Y, Tashiro K, Suematsu Y, Kuwano T, Kitajima K, Saku K, Miura SI (2019). Association between plasma levels of PCSK9 and the presence of coronary artery disease in Japanese. Heart Vessels.

[13] Chan DC, Pang J, McQuillan BM, Hung J, Beilby JP, Barrett PH, Watts GF (2016). Plasma proprotein convertase subtilisin kexin type 9 as a predictor of carotid atherosclerosis in asymptomatic adults. Heart Lung Circ..

[14] Lee CJ, Lee YH, Park SW, Kim KJ, Park S, Youn JC, Lee SH, Kang SM, Jang Y (2013). Association of serum proprotein convertase subtilisin/kexin type 9 with carotid intima media thickness in hypertensive subjects. Metabolism..

[15] Tóth Š, Fedačko J, Pekárová T, Hertelyová Z, Katz M, Mughees A, Kuzma J, Štefanič P, Kopolovets I, Pella D (2017). Elevated circulating PCSK9 concentrations predict subclinical atherosclerotic changes in low risk obese and non-obese patients. Cardiol Ther..

[16] Alonso R, Mata P, Muñiz O, Fuentes-Jimenez F, Díaz JL, Zambón D, Tomás M, Martin C, Moyon T, Croyal M, Thedrez A, Lambert G (2016). PCSK9 and lipoprotein (a) levels are two predictors of coronary artery calcification in asymptomatic patients with familial hypercholesterolemia. Atherosclerosis..

[17] Zhao X, Zhang HW, Li S, Zhang Y, Xu RX, Zhu CG, Wu NQ, Guo YL, Qing P, Li XL, Liu G, Dong Q, Sun J, Li JJ (2018). Association between plasma proprotein convertase subtisilin/kexin type 9 concentration and coronary artery calcification. Ann Clin Biochem.

[18] Shapiro MD, Tavori H, Fazio S (2018). PCSK9: from basic science discoveries to clinical trials. Circ Res.

[19] Pasterkamp G, den Ruijter HM, Libby P (2017). Temporal shifts in clinical presentation and underlying mechanisms of atherosclerotic disease. Nat Rev Cardiol..

[20] Libby P, Buring JE, Badimon L, Hansson GK, Deanfield J, Bittencourt MS, Tokgözoğlu L, Lewis EF (2019). Atherosclerosis. Nat Rev Dis Primers..

[21] Neglia D, Rovai D, Caselli C, EVINCI Study Investigators (2015). Detection of significant coronary artery disease by noninvasive anatomical and functional imaging. Circ Cardiovasc Imaging..

[22] Rovai D, Neglia D, Lorenzoni V, Caselli C, Knuuti J, Underwood SR, EVINCI Study Investigators (2015). Limitations of chest pain categorization models to predict coronary artery disease. Am J Cardiol..

[23] Caselli C, De Graaf MA, Lorenzoni V, Rovai D, Marinelli M, Del Ry S, Giannessi D, Bax JJ, Neglia D, Scholte AJ (2015). HDL cholesterol, leptin and interleukin-6 predict high risk coronary anatomy assessed by CT angiography in patients with stable chest pain. Atherosclerosis..

[24] Caselli C, Rovai D, Lorenzoni V, EVINCI Study Investigators (2015). A new integrated clinical-biohumoral model to predict functionally significant coronary artery disease in patients with chronic chest pain. Can J Cardiol..

[25] Caselli C, Prontera C, Liga R (2016). Effect of coronary atherosclerosis and myocardial ischemia on plasma levels of high-sensitivity troponin T and NT-proBNP in patients with stable angina. Arterioscler Thromb Vasc Biol.

[26] Liga R, Vontobel J, Rovai D, EVINCI Study Investigators (2016). Multicentre multi-device hybrid imaging study of coronary artery disease: results from the EValuation of INtegrated Cardiac Imaging for the Detection and Characterization of Ischaemic Heart Disease (EVINCI) hybrid imaging population. Eur Heart J Cardiovasc Imaging..

[27] de Graaf MA, Broersen A, Ahmed W, Kitslaar PH, Dijkstra J, Kroft LJ, Delgado V, Bax JJ, Reiber JH, Scholte AJ (2014). Feasibility of an automated quantitative computed tomography angiography-derived risk score for risk stratification of patients with suspected coronary artery disease. Am J Cardiol.

[28] van Rosendael AR, Shaw LJ, Xie JX (2019). Superior risk stratification with coronary computed tomography angiography using a comprehensive atherosclerotic risk score. JACC Cardiovasc Imaging..

[29] Matthews DR, Hosker JP, Rudenski AS, Naylor BA, Treacher DF, Turner RC (1985). Homeostasis model assessment: insulin resistance and beta-cell function from fasting plasma glucose and insulin concentrations in man. Diabetologia.

[30] WHO (2000). Obesity preventing and managing the global epidemic. Report of a WHO Consultation.

[31] Grundy SM, Cleeman JI, Daniels SR, Donato KA, Eckel RH, Franklin BA, Gordon DJ, Krauss RM, Savage PJ, Smith SC, Spertus JA, Costa F, American Heart Association; National Heart, Lung, and Blood Institute (2005). Diagnosis and management of the metabolic syndrome: an American Heart Association/National Heart, Lung, and Blood Institute Scientific Statement. Circulation..

[32] Alberti KG, Zimmet P, Shaw J, IDF Epidemiology Task Force Consensus Group (2005). The metabolic syndrome—a new worldwide definition. Lancet..

[33] Friedewald WT, Levy RI, Fredrickson DS (1972). Estimation of the concentration of low-density lipoprotein cholesterol in plasma, without use of the preparative ultracentrifuge. Clin Chem.

[34] Filippatos TD, Kei A, Rizos CV, Elisaf MS (2018). Effects of PCSK9 inhibitors on other than low-density lipoprotein cholesterol lipid variables. J Cardiovasc Pharmacol Ther..

[35] Lakoski SG, Lagace TA, Cohen JC, Horton JD, Hobbs HH (2009). Genetic and metabolic determinants of plasma PCSK9 levels. J Clin Endocrinol Metab.

[36] Cui Q, Ju X, Yang T, Zhang M, Tang W, Chen Q, Hu Y, Haas JV, Troutt JS, Pickard RT, Darling R, Konrad RJ, Zhou H, Cao G (2010). Serum PCSK9 is associated with multiple metabolic factors in a large Han Chinese population. Atherosclerosis..

[37] Dubuc G, Tremblay M, Paré G, Jacques H, Hamelin J, Benjannet S, Boulet L, Genest J, Bernier L, Seidah NG, Davignon J (2010). A new method for measurement of total plasma PCSK9: clinical applications. J Lipid Res.

[38] Ferri N, Corsini A, Macchi C, Magni P, Ruscica M (2016). Proprotein convertase subtilisin kexin type 9 and high-density lipoprotein metabolism: experimental animal models and clinical evidence. Transl Res..

[39] Victor RG, Haley RW, Willett DL, Peshock RM, Vaeth PC, Leonard D, Basit M, Cooper RS, Iannacchione VG, Visscher WA, Staab JM, Hobbs HH, Dallas Heart Study Investigators (2004). The Dallas Heart Study: a population-based probability sample for the multidisciplinary study of ethnic differences in cardiovascular health. Am J Cardiol..

[40] Abifadel M, Guerin M, Benjannet S (2012). Identification and characterization of new gain-of-function mutations in the PCSK9 gene responsible for autosomal dominant hypercholesterolemia. Atherosclerosis..

[41] Aung LH, Yin RX, Miao L, Hu XJ, Yan TT, Cao XL, Wu DF, Li Q, Pan SL, Wu JZ (2011). The proprotein convertase subtilisin/kexin type 9 gene E670G polymorphism and serum lipid levels in the Guangxi Bai Ku Yao and Han populations. Lipids Health Dis..

[42] Werner C, Hoffmann MM, Winkler K, Böhm M, Laufs U (2014). Risk prediction with proprotein convertase subtilisin/kexin type 9 (PCSK9) in patients with stable coronary disease on statin treatment. Vascul Pharmacol.

[43] Careskey HE, Davis RA, Alborn WE, Troutt JS, Cao G, Konrad RJ (2008). Atorvastatin increases human serum levels of proprotein convertase subtilisin/kexin type 9. J Lipid Res.

[44] Mayne J, Dewpura T, Raymond A, Cousins M, Chaplin A, Lahey KA, Lahaye SA, Mbikay M, Ooi TC, Chrétien M (2008). Plasma PCSK9 levels are significantly modified by statins and fibrates in humans. Lipids Health Dis..

[45] Raal F, Panz V, Immelman A, Pilcher G (2013). Elevated PCSK9 levels in untreated patients with heterozygous or homozygous familial hypercholesterolemia and the response to high-dose statin therapy. J Am Heart Assoc..

[46] Ferri N, Ruscica M (2016). Proprotein convertase subtilisin/kexin type 9 (PCSK9) and metabolic syndrome: insights on insulin resistance, inflammation, and atherogenic dyslipidemia. Endocrine.

[47] Awan Z, Dubuc G, Faraj M, Dufour R, Seidah NG, Davignon J, Rabasa-Lhoret R, Baass A (2014). The effect of insulin on circulating PCSK9 in postmenopausal obese women. Clin Biochem.

[48] Ference BA, Robinson JG, Brook RD, Catapano AL, Chapman MJ, Neff DR, Voros S, Giugliano RP, Davey Smith G, Fazio S, Sabatine MS (2016). Variation in PCSK9 and HMGCR and risk of cardiovascular disease and diabetes. N Engl J Med.

[49] Schmidt AF, Swerdlow DI, Holmes MV (2017). UCLEB consortium, Sattar N. PCSK9 genetic variants and risk of type 2 diabetes: a mendelian randomisation study. Lancet Diabetes Endocrinol..

[50] Lotta LA, Sharp SJ, Burgess S (2016). Association between low-density lipoprotein cholesterol-lowering genetic variants and risk of type 2 diabetes: a meta-analysis. JAMA.

[51] Yang SH, Xu RX, Cui CJ, Wang Y, Du Y, Chen ZG, Yao YH, Ma CY, Zhu CG, Guo YL, Wu NQ, Sun J, Chen BX, Li JJ (2018). Liraglutide downregulates hepatic LDL receptor and PCSK9 expression in HepG2 cells and db/db mice through a HNF-1a dependent mechanism. Cardiovasc Diabetol..

[52] de Carvalho LSF, Campos AM, Sposito AC (2018). Proprotein convertase subtilisin/kexin type 9 (PCSK9) inhibitors and incident type 2 diabetes: a systematic review and meta-analysis with over 96,000 patient-years. Diabetes Care.

[53] Saavedra YGL, Dufour R, Baass A (2015). Familial hypercholesterolemia: PCSK9 InsLEU genetic variant and prediabetes/diabetes risk. J Clin Lipidol..

[54] Awan Z, Delvin EE, Levy E, Genest J, Davignon J, Seidah NG, Baass A (2013). Regional distribution and metabolic effect of PCSK9 insLEU and R46L gene mutations and apoE genotype. Can J Cardiol.

[55] Paige E, Masconi KL, Tsimikas S, Kronenberg F, Santer P, Weger S, Willeit J, Kiechl S, Willeit P (2017). Lipoprotein(a) and incident type-2 diabetes: results from the prospective Bruneck study and a meta-analysis of published literature. Cardiovasc Diabetol..

[56] Satish M, Saxena SK, Agrawal DK (2019). Adipokine dysregulation and insulin resistance with atherosclerotic vascular disease: metabolic syndrome or independent sequelae?. J Cardiovasc Transl Res..

[57] Ruscica M, Ferri N, Macchi C, Meroni M, Lanti C, Ricci C, Maggioni M, Fracanzani AL, Badiali S, Fargion S, Magni P, Valenti L, Dongiovanni P (2016). Liver fat accumulation is associated with circulating PCSK9. Ann Med.

[58] Baass A, Dubuc G, Tremblay M, Delvin EE, O’Loughlin J, Levy E, Davignon J, Lambert M (2009). Plasma PCSK9 is associated with age, sex, and multiple metabolic markers in a population-based sample of children and adolescents. Clin Chem.

[59] Sookoian S, Pirola CJ (2015). Liver enzymes, metabolomics and genome-wide association studies: from systems biology to the personalized medicine. World J Gastroenterol.

[60] Quispe R, Elshazly MB, Zhao D, Toth PP, Puri R, Virani SS, Blumenthal RS, Martin SS, Jones SR, Michos ED (2019). Total cholesterol/HDL-cholesterol ratio discordance with LDL-cholesterol and non-HDL-cholesterol and incidence of atherosclerotic cardiovascular disease in primary prevention: the ARIC study. Eur J Prev Cardiol..

