# Supplementary material for: Association of PCSK9 plasma levels with metabolic patterns and coronary atherosclerosis in patients with stable angina
Source: Cardiovasc Diabetol. 2019 Oct 31;18:144. doi: 10.1186/s12933-019-0949-3 (PMC6824037; doi:10.1186/s12933-019-0949-3)
Supplement: Supplementary file 1 — Additional file 1. Additional tables. [file 12933_2019_949_MOESM1_ESM.pdf]

ADDITIONAL FILE 1.

Table S1: Clinical characteristics of study population relative to PCSK9 quartiles

	Total Population n=539	Quartile I <138 ng/L n=135	Quartile II 138-183.8 ng/L n=135	Quartile III 183.9-264 ng/L n=135	Quartile IV >264 ng/L n=134	P value
**Demographics**						
Age, years	60±9	61±9	58±10	62±8	61±8	ns
Male gender	326 (60)	88 (65)	82 (61)	84 (62)	72 (58)	ns
**Clinical characteristics**						
Typical angina	140 (26)	30 (22)	34 (25)	32 (24)	44 (33)	
Atypical angina	321 (60)	78 (58)	87 (64)	79 (58)	77 (57)	
Non-anginal chest pain	78 (14)	27 (20)	14 (10)	24 (18)	13 (10)	
LVEF%	60±8	60±9	60±9	59±9	61±7	ns
Pre-test probability of CAD	48±19	48±18	47±19	49±19	49±20	ns
**Cardiovascular risk factors**						
Family history of CAD	189 (35)	40 (30)	48 (36)	42 (31)	59 (44)	0.0596
Diabetes	181 (34)	44 (33)	53 (39)	45 (33)	39 (29)	ns
Hypercholesterolemia	322 (60)	77 (57)	82 (61)	81 (60)	82 (61)	ns
Hypertension	360 (67)	88 (65)	93 (69)	88 (65)	91 (68)	ns
Smoking	133 (25)	30 (22)	42 (31)	27 (20)	34 (25)	ns
BMI, kg/m^2	27.7±4.3	27.9±4.0	28.2±4.1	27.7±4.5	26.8±4.6	ns
Metabolic Syndrome	185 (34)	54 (40)	54 (40)	46 (34)	31 (23)	0.0102
**Pharmacological therapies**						
Beta-blockers	215 (40)	64 (47)	51 (38)	54 (40)	46 (34)	ns
Calcium channel blockers	74 (14)	21 (16)	16 (12)	16 (12)	21 (15)	ns
ACE Inhibitors	166 (31)	43 (32)	39 (29)	48 (36)	36 (27)	ns
ARBs	91 (17)	23 (17)	26 (19)	27 (13)	25 (19)	ns
Diuretics	93 (17)	27 (20)	24 (18)	20 (15)	22 (16)	ns
Anti-diabetic	98 (18)	25 (19)	30 (22)	25 (18)	18 (13)	ns
Statins	279 (52)	72 (53)	77 (57)	71 (53)	59 (44)	ns
Aspirin	316 (59)	94 (70)	66 (49)	81 (60)	75 (56)	0.0058
Anti-coagulants	11 (2)	2 (1)	3 (2)	2 (1)	4 (3)	ns

Continuous variables are presented as mean ± standard deviation, categorical variables as absolute N and (%).

Table S2. Bio-humoral characteristics of clinical population relative to PCSK9 quartiles.

	Total Population n=412	Quartile I <136 ng/L n=103	Quartile II 136-183.8 ng/L n=206	Quartile III 183.9-264 ng/L n=206	Quartile IV >264 ng/L n=103	P value
**Renal**						
Creatinine, mg/dL	0.88±0.23	0.91±0.27	0.87±0.24	0.88±0.21	0.86±0.19	ns
**Hepatic**						
AST, IU/L	24±10	24±11	23±7	26±11	26±9	0.0062
ALT, IU/L	21±13	19±11	19±11	22±16	22±13	0.0111
ALP, IU/L	51±18	47±17	47±17	51±15	60±20	<0.0001
**Metabolic (glucose)**						
FPG, mg/dL	112±35	109±30	117±43	116±38	109±29	ns
†FPG ≥ 100 mg/dL	339 (63)	82 (61)	81 (60)	93 (69)	83 (62)	ns
Insulin, µUI/mL	11.5±10.8	13.3±12.5	12.6±12.2	10.0±7.2	10.3±10.1	0.0344*
HOMA index	3.4±4.1	3.9±4.5	4.0±5.2	2.9±2.6	2.9±3.2	0.0566*
**Metabolic (lipid)**						
Total cholesterol, mg/dL	183±49	171±43	179±46	182±44	201±55	<0.0001
LDL, mg/dL	106±40	100±37	103±38	105±38	118±45	0.0043
HDL, mg/dL	53±17	47±13	51±16	52±14	61±19	<0.0001
†HDL ≤ 40 mg/dL, male or HDL ≤ 50 mg/dL, female	160 (30)	60 (44)	47 (34)	34 (26)	19 (14)	<0.0001
Apo A1, mg/dL	143±32	132±32	139±32	148±27	155±35	<0.0001
Apo B, mg/dL	87±28	82±28	86±26	88±27	93±29	0.0048
Lipoprotein (a)	21.3±23.2	16.1±18.3	21.6±21.6	22.9±22.0	24.6±26.5	0.0052
Tryglicerides, mg/dL	124±81	125±85	127±83	127±89	118±68	ns
†Tryglicerides ≥150 mg/dL	131 (24)	34 (25)	36 (25)	31 (23)	30 (23)	ns
**Inflammatory and vascular**						
hs-CRP, mg/dL	0.40±1.09	0.41±0.61	0.30±0.34	0.47±1.89	0.43±0.84	ns
Interleukin 6, ng/L	1.35±2.53	1.56±2.71	1.10±1.25	1.46±3.24	1.30±1.67	ns
**Adipokines**						
Adiponectin, µg/mL	9.81±6.92	8.11±5.06	10.21±8.04	9.74±6.28	11.19±7.62	0.0019
Leptin, ng/mL	9.93±10.66	11.38±13.01	9.06±8.89	9.27±9.15	9.99±10.98	ns

Continuous variables are presented as mean ± standard deviation, categorical variables as absolute N and (%); * I vs. IV quartile; † criteria of Metabolic Syndrome.

Table S3. Angiography imaging results relative to PCSK9 groups.

	CTA Population (n=412)	Quartile I <136 ng/L n=103	Quartile II-III 136-183.8 ng/L n=103	Quartile III 183.9-266 ng/L n=103	Quartile IV >266 ng/L n=103	P value
**Obstructive CAD at CTA**						
Stenosis >50%	144 (35)	40 (39)	31 (30)	40 (38)	33 (32)	ns
Stenosis >70%	45 (11)	12 (12)	12 (12)	11 (11)	10 (10)	ns
**Coronary plaques**						
Total N. of plaques	3.9±3.8	4.36±3.89	3.70±3.63	4.23±3.90	3.37±3.77	0.0621*
N. of non-obstructive plaques	3.03±3.0	3.33±2.89	2.96±2.89	3.14±3.31	2.43±2.93	0.0323*
N. of obstructive plaques	0.9±1.7	0.94±1.57	0.63±1.43	0.84±1.43	0.85±1.87	ns
N. of calcified plaques	0.9±1.7	0.85±1.84	0.83±1.42	1.04±1.93	0.81±1.74	ns
N. of mixed plaques	2.6±3.2	3.14±3.42	2.45±3.14	2.54±3.14	2.19±3.15	0.0358*
N. of non-calcified plaques	0.5±0.9	0.38±0.85	0.43±0.78	0.66±1.21	0.38±0.72	ns
**CTA risk score**	11.71±11.05	13.17±11.53	10.88±10.43	12.77±10.95	9.99±11.11	0.0390*

Continuous variables are presented as mean ± standard deviation, categorical variables as absolute N and (%); * I vs. IV quartile.

Table S4: Univariate analysis of the association between clinical variables and CTA risk score

	Univariate analysis	
	**Coefficient (SE)**	**P Value**
**Age**	0.067 (0.007)	<0.0001
**Male gender**	0.774 (0.131)	<0.0001
**Typical angina**	-0.340 (0.155)	0.0288
**LVEF**	-0.022 (0.008)	0.0059
**Pre-test probability of CAD**	0.005 (0.003)	0.0609
**Family history of CAD**	-0.298 (0.139)	0.0326
**Diabetes**	0.458 (0.152)	0.0028
**Hypercholesterolemia**	0.157 (0.134)	ns
**Hypertension**	0.478 (0.133)	0.0004
**Smoking**	0.074 (0.152)	ns
**BMI**	0.016 (0.017)	ns
**Metabolic Syndrome**	0.373 (0.152)	0.0143
**Beta-blockers**	0.386 (0.134)	0.0041
**Calcium channel blockers**	0.380 (0.203)	ns
**ACE Inhibitors**	0.355 (0.145)	0.0146
**ARBs**	0.386 (0.179)	0.0318
**Diuretics**	-0.269 (0.178)	ns
**Anti-diabetic**	0.0465 (0.167)	0.0056
**Statins**	0.543 (0.130)	<0.0001
**Aspirin**	0.535 (0.131)	<0.0001
**Anti-coagulants**	0.670 (0.156428	ns

Table S5: Univariate analysis of the association between biohumoral variables and CTA risk score

	Univariate analysis	
	**Coefficient (SE)**	**P Value**
**Creatinin**	1.069 (0.599)	0.0750
**AST**	0.036 (0.186)	ns
**ALT**	-0.119 (0.138)	ns
**ALP**	-0.083 (0.185)	ns
**Glicemia**	0.514 (0.259)	0.0475
**Insulin**	0.249 (0.086)	0.0041
**HOMA index**	0.322 (0.107)	0.0029
**Total cholesterol**	-0.859 (0.244)	0.0005
**LDL cholesterol**	-0.519 (0.166)	0.0019
**HDL cholesterol**	-0.829 (0.207)	< 0.0001
**Apo A1**	-0.911 (0.253)	0.0004
**Apo B**	-0.308 (0.202)	ns
**Lipoprotein (a)**	0.016 (0.065)	ns
**Triglycerides**	0.143 (0.121)	ns
**hs-CRP**	0.0152 (0.036)	ns
**Interleukin-6**	0.496 (0.137)	0.0003
**Adiponectin**	-0.118 (0.105)	ns
**Leptin**	-0.318 (0.073)	<0.0001
**PCSK9**	-0.277 (0.135)	0.0408

Table S6. Univariate and multivariate analyses of the association between PCSK9 and statins or all drugs.

	**PCSK9**	
	Coefficient± SE	P
**AST**	0.202±0.056	0.0004
**AST + statins**	0.201±0.056	0.0004
**AST + all drugs**	0.205±0.057	0.0003
	Coefficient± SE	P
**ALT**	0.142±0.042	0.0009
**ALT + statins**	0.143±0.042	0.0008
**ALT + all drugs**	0.142±0.042	0.0008
	Coefficient± SE	P
**ALP**	0.389±0.055	0.0001
**ALP + statins**	0.387±0.056	<0.0001
**ALP + all drugs**	0.389±0.055	<0.0001

Table S7. Univariate and multivariate analyses of the association among PCSK9 and hepatic enzymes, risk factors, CTA risk score.

	PCSK9	
	Coefficient± SE	P
**AST**	0.202±0.056	0.0004
**AST + risk factors***	0.205±0.056	0.0003
**AST + CTA score**	0.183±0.066	0.0059
	Coefficient± SE	P
**ALT**	0.142±0.042	0.0009
**ALT + risk factors***	0.157±0.043	0.0002
**ALT + CTA score**	0.131±0.050	0.0085
	Coefficient± SE	P
**ALP**	0.389±0.055	0.0001
**ALP + risk factors***	0.372±0.057	<0.0001
**ALP + CTA score**	0.389±0.064	<0.0001

*Risk factors included Diabetes, Hypecholesterolemia, Hypertriglyceridemia, Obesity, and Metabolic Syndrome
